# What’s new in atopic eczema? An analysis of systematic reviews published in 2018. Part 1: prevention and topical therapies

**DOI:** 10.1111/ced.14303

**Published:** 2020-08-27

**Authors:** F. Tasker, A. Brown, D. J. C. Grindlay, N. K. Rogers, K. E. Harman

**Affiliations:** ^1^ Guy’s and St Thomas’ Hospitals NHS Foundation Trust London UK; ^2^ University Hospital Plymouth NHS Trust Devon UK; ^3^ Centre of Evidence Based Dermatology University of Nottingham King’s Meadow Campus Nottingham UK

## Abstract

This review is part of a series of annual updates that summarize the evidence base for atopic eczema (AE). The aim is to provide a succinct guide for clinicians on the key findings from 14 systematic reviews on the prevention and topical treatment of AE published or indexed in 2018. Various supplements, including long‐chain polyunsaturated fatty acids, vitamin D and the probiotic *Lactobacillus rhamnosus* GG, given prenatally and postnatally, have not been shown to prevent AE in infants, although mixed strains of probiotics may decrease the risk of AE if given to the mother during pregnancy and to the infant for the first 6 months of life. In the postnatal period, there is no evidence that hydrolysed formula, compared with cow’s milk formula (CMF), reduces the risk of AE in partially breastfed infants. However, weak evidence suggests that a specific partially hydrolysed whey formula decreases the risk of AE compared with CMF. No specific skin practices can be recommended to reduce the eczema risk in healthy term babies. There is weak evidence of a low risk of reversible hypothalamic–pituitary–adrenal axis suppression following 2–4 weeks of treatment with low‐potency topical steroids, and conflicting evidence as to whether bleach bathing affects skin flora or AE severity. A single study demonstrated that the topical Janus kinase inhibitor tofacitinib at 2% significantly reduces the Eczema Area and Severity Index compared with vehicle. Topical naltrexone cream 1% improves pruritus (measured using a visual analogue scale) by 30% more than placebo. There is weak evidence that topical alternative therapies, including antioxidants, micronutrients and some herbal medicines, may improve AE.

## Abstract 2

Click here for the corresponding questions to this CME article.

## Background

The aim of this two‐part evidence update is to present key findings from systematic reviews (SRs) published or indexed in 2018, summarizing prevention of atopic eczema (AE) and treatment with topical therapies. Part 2 will cover systemic therapies. The details of the search methods can be found at: https://www.nottingham.ac.uk/research/groups/cebd/documents/methodological‐resources/ebu‐protocol.pdf.

## Prevention of atopic eczema

### Prenatal prevention

#### Long chain polyunsaturated fatty acids

A meta‐analysis of six randomized controlled trials (RCTs), involving 1861 infants, found no evidence supporting prenatal intake of long‐chain polyunsaturated fatty acids for the prevention of AE.^1^ The included studies were at high risk of bias and moderately heterogeneous, and included infants with high and low risk of atopy. It was unclear how AE was defined, and there was variation in dose and timing of supplementation.

### Prenatal and postnatal prevention

#### Probiotics

In infants at high risk of allergy, the World Allergy Organization recommends probiotics for the mother during pregnancy and when breastfeeding and for the infants.^2^ Although a meta‐analysis found that the probiotic *Lactobacillus rhamnosus* GG (LGG) given prenatally and/or postnatally did not reduce the risk of AE,^3^ this meta‐analysis only included a small number of studies (5 RCTs, 889 participants), which had significant heterogeneity, and only LGG supplementation was evaluated.

Another review looked at a larger number of studies (27 RCTs and 1 controlled cohort study, 6907 participants) and concluded that mixtures of probiotics, including *Lactobacillus*, *Bifidobacterium* and *Propionibacterium* strains, significantly decrease the risk of AE when supplementation starts during pregnancy and continues in the infant through the first 6 months postnatally (OR = 0.69, 95% CI 0.54–0.82. *P* < 0.0001).^4^ However, it is not clear whether probiotic supplementation in the first 6 months of life was via the mother or directly to the infant by mouth. There was also significant study heterogeneity, with differing definitions for AE and varying probiotic strains used.

#### Vitamin D

A review on vitamin D concluded there was no evidence to support supplementation during pregnancy, breastfeeding or early infant life for the prevention of allergic diseases.^5^ One RCT (151 pregnant women) showed no benefit of vitamin D supplementation for primary prevention of AE in children (risk ratio 0.96, 95% CI 0.57–1.61). Overall, the quality of evidence was low.

### Postnatal prevention

#### Hydrolysed protein formula in infants

A high‐quality Cochrane Review concluded that there was no evidence for early, short‐term (3–4 days) feeding of infants with hydrolysed formula compared with exclusive breastfeeding to prevent eczema (*n* = 90).^6^ Additionally, no evidence was found for the use of short‐term (3–4 days, *n* = 77) or prolonged (in the first months of life) (*n* = 2896) feeding with hydrolysed formula, compared with cow’s milk formula (CMF), for the prevention of AE in infants who were partially breastfed.^6^ This was in contrast to a review, which concluded that, compared with CMF, a specific, partially hydrolysed whey infant formula decreased the risk of AE at 12 months of age in partially breastfed infants from the general population (OR = 0.6, 95% CI 0.45–0.80).^7^ Many of the studies in this latter review were of low quality and had a high risk of bias.

#### Hydrolysed protein formula in infants

Skin‐care practices, such as bathing, cleansing, nappy care and the management of dry skin with emollients, used for healthy term babies were investigated in a review that included 26 studies and 16 RCTs.^8^ A wide range of interventions (*n* = 13) were included and although they did not measure eczema directly, primary outcomes evaluated change in stratum corneum hydration, transepidermal water loss and skin surface pH within 6 months of birth. There was no evidence of significant differences between the tested wash products or wipes compared with water. Two RCTs examined the impact of daily application of an emollient in neonates at risk of AE (*n* = 118 and *n* = 124). Although both studies suggested that daily emollient application from birth reduced the risk of AE at 6 months, the evidence from one of these was weak, being a pilot trial assessing feasibility of the approach. Follow‐up in studies in this SR was variable and short (days to weeks or a few months, although one study was assessed at 24 months), and many of the studies had a significant risk of bias.

## Topical therapies for atopic eczema

### Side effects of topical corticosteroid

The likelihood of hypothalamic–pituitary–adrenal (HPA) axis suppression following 2–4 weeks of treatment with topical corticosteroid (TCS) for patients aged ≤ 18 years with AE was reviewed (11 open‐label studies, 1 randomized open‐label study).^9^ Although 20 (3.8%, 95% CI 2.4–5.8) of the 522 cases had HPA axis suppression, this may be an overestimate, as the adrenocorticotropic hormone stimulation test used has been criticized for yielding false positives. The risk was lower in children using low‐potency TCS. The overall confidence level of this meta‐analysis was critically low, and the studies included had significant heterogeneity with regard to TCS duration, dose and application site. TCS are typically used for ≥ 4 weeks, so studies with longer term follow‐up are required.

A well‐designed SR intended to explore the risk of skin cancer with long‐term use of TCS (more than once a week for ≥ 1 month); however, the authors were unable to answer the question because no suitable studies were identified.^10^

### Efficacy of bleach baths

A review of bleach baths assessed 14 studies (prospective cohort studies and RCTs) in adults and children.^11^ Compared with water baths, the evidence for their effect on the severity of AE and on the skin flora was conflicting. Studies were limited by poor quality, small patient numbers and high risk of bias. The effect of bleach baths alone was unclear due to use of combination treatments. A high‐quality RCT is needed to assess this issue.

### Janus kinase inhibitors

Two reviews from the same group in different years assessed the applications of Janus kinase inhibitors (JAK‐I) in skin diseases.^12^, ^13^ The reviews included a single RCT for AE.^12^, ^13^ The study evaluated the use of 2% tofacitinib against vehicle for 69 adult participants over a 4‐week period. Reduction in Eczema Area and Severity Index (EASI) was significantly greater for tofacitinib (81.7%) than for vehicle (29.9%). The proportion of patients ‘clear’ or ‘almost clear’ was 73% for tofacitinib and 22% for vehicle, and pruritus was significantly reduced with tofacitinib compared with vehicle. Future studies need to have longer follow‐up and use active standard care comparators.

### Topical naltrexone 1%

A review of 22 articles included one RCT on topical naltrexone 1% with crossover of 40 adults with localized and generalized AE with severe pruritus, and showed that the treatment improved pruritus visual analogue scores by 30% more than a vehicle placebo.^14^

### Topical antioxidants

A drug company‐sponsored review of emollients containing furfuryl palmitate and furfuryl derivatives concluded that these compounds were safe and effective for mild to moderate AE.^15^ The included studies were of a short duration (14–30 days), the instruments used to assess outcomes were unclear, and some products contained multiple agents. Although claims were made relative to steroid efficacy, only one of the six studies (*n* = 40) compared furpalmate against topical steroids; the others were compared against vehicle or placebo, or had no comparison group. These limitations make it difficult to draw a valid conclusion from this SR.

### Topical micronutrients

An SR summarized the current evidence for topical micronutrient application in AE. It reported that certain vitamins (B, C and E) and minerals (magnesium, zinc and iodine) appeared to provide some benefit, although vitamin A was not effective and vitamin D may have exacerbated symptoms. The 16 studies had varied methodology, small sample sizes, and included five murine studies, a case report, a case–control study, cross‐sectional studies, split‐body comparison studies and RCTs in 571 participants with AE (including children and adults). The outcomes were mixed, and had time frames for treatment ranging from 3 days to 8 weeks, making comparison difficult. A standardized outcome such as SCORing Atopic Dermatitis (SCORAD) was used in only three studies. It is difficult to draw valid conclusions from this SR.

### *Mahonia aquifolium*

An SR evaluated *Mahonia aquifolium* (Oregon grape), a plant used in traditional Chinese medicine, for skin conditions.^16^ Only one single‐arm efficacy study was included, involving 42 adults with AE, which assessed a cream containing 10% proprietary *M. aquifolium*. Although a statistically significant difference in EASI was observed at 12 weeks treatment compared with baseline (2.01 reduced down to 0.06), baseline severity was very low and the mean reduction failed to reach the minimum clinically important difference of 6.6.^16^

**Learning points**

- There is no evidence that long chain polyunsaturated fatty acids, vitamin D and the probiotic L* *GG reduce the risk of AE when given during pregnancy, although probiotic mixtures may decrease the risk of AE if given in pregnancy and for the first 6 months of the infant’s life.
- In the postnatal period, there is no evidence for the use of hydrolysed formula compared with CMF for the prevention of eczema in infants who are not exclusively breastfed.
- There is conflicting evidence as to whether bleach baths, compared with water baths, have any effect on the skin flora and severity of AE.
- Topical tofacitinib 2% (a JAKi) improves the severity of AE compared with vehicle.
- There is weak evidence for the use of topical alternative therapies to improve AE, including antioxidants, micronutrients and *M. aquifolium*.

## CPD questions

### Learning objective

To demonstrate up‐to‐date knowledge of prevention and treatment for atopic eczema.

### Question 1

Which of the following statements regarding the use of probiotics in atopic eczema (AE) is true?

- The World Allergy Organization does not recommend using probiotics in pregnant women at high risk for having an allergic child.
- There is meta‐analysis evidence that administration of *Lactobacillus rhamnosus* GG during the prenatal and/or postnatal period for reduces the risk of AE in infants.
- There is some evidence to suggest that mixtures of *Lactobacillus*, *Bifidobacterium* and *Propionibacterium* strains significantly decrease the risk of AE when started during the mother’s pregnancy and continued through the first 6 months of the infant’s life.
- Randomized controlled trials investigating the use of probiotics are consistent in their definition of AE.
- It is easy to compare studies of probiotics in AE owing to consistency in dose and strain.

### Question 2

Which of the following statements regarding skin care in the prevention of atopic eczema (AE) is true?

- Transepidermal water loss is an invasive *in vivo* measurement of water loss across the stratum corneum.
- Transepidermal water loss is a direct measure of AE.
- There is no meta‐analysis evidence that certain wash products or baby wipes increase the risk of AE compared with water.
- There is strong evidence that daily emollient application from birth prevents AE.
- One of the strengths of studies in skin care in AE is the length of follow‐up.

### Question 3

Which of the following statements regarding the prevention of atopic eczema (AE) is correct?

- A meta‐analysis showed evidence that intake of long‐chain polyunsaturated fatty acids in breastfeeding women was better than placebo for the prevention of AE in infants.
- A meta‐analysis showed no evidence that prenatal intake of long‐chain polyunsaturated fatty acids was better than placebo for the prevention of AE in infants, but there was moderate heterogeneity and high risk of bias in included studies.
- A systematic review showed that the probiotic *Lactobacillus rhamnosus* GG reduced the risk of AE in infants when given during the mother’s pregnancy.
- A systematic review outlined evidence to support that pregnant and breastfeeding women should take vitamin D supplements to reduce the risk of AE in their offspring.
- A systematic review outlines evidence to suggest infants should be given vitamin D supplements to reduce the risk of AE.

### Question 4

Which of the following statements is correct regarding the potential adverse effects of topical corticosteroid (TCS) for patients aged ≤ 18 years or with atopic eczema (AE) ?

- Skin cancer is associated with long‐term use of TCS.
- The adrenocorticotropic hormone stimulation test is a foolproof investigation for assessing hypothalamic–pituitary–adrenal (HPA) axis suppression.
- There is a high rate of HPA axis suppression following 2–4 weeks of treatment with TCS for patients aged ≤ 18 years with AE.
- There is a low risk of HPA axis suppression following 2–4 weeks of treatment with TCS for patients aged ≤ 18 years with AE.
- Children using low‐potency TCS have a high risk of reversible HPA axis suppression.

### Question 5

Which of the following statements regarding topical treatments for atopic eczema (AE) is true?

- The topical Janus kinase inhibitor (JAKi) tofacitinib given for AE has been shown to produce a significantly greater percentage change than vehicle in Eczema Area and Severity Index (EASI) after 4 weeks.
- A systemic review including studies of high quality showed evidence that furfuryl palmitate and furfuryl derivatives added to an emollient formulation in mild to moderate AE were effective.
- A systematic review including studies of high quality concluded that formulations of vitamins B, C and E and the minerals magnesium, zinc and iodine improve AE in children.
- A systematic review concluded that topical vitamin A and D improved AE in children and adults.
- A systematic review including studies of high quality concluded that topical *Mahonia aquifolium* significantly reduces EASI in AE.

## Instructions for answering questions

This learning activity is freely available online at http://www.wileyhealthlearning.com/ced

Users are encouraged to

- Read the article in print or online, paying particular attention to the learning points and any author conflict of interest disclosures
- Reflect on the article
- Register or login online at http://www.wileyhealthlearning.com/ced and answer the CPD questions
- Complete the required evaluation component of the activity

Once the test is passed, you will receive a certificate and the learning activity can be added to your RCP CPD diary as a self‐certified entry.

This activity will be available for CPD credit for 2 years following its publication date. At that time, it will be reviewed and potentially updated and extended for an additional period.
